# Hemodialysis vascular access and subsequent transplantation: a report from the ESPN/ERA-EDTA Registry

**DOI:** 10.1007/s00467-018-4129-6

**Published:** 2018-12-26

**Authors:** Michael Boehm, Marjolein Bonthuis, Marlies Noordzij, Jérôme Harambat, Jaap W. Groothoff, Ángel Alonso Melgar, Jadranka Buturovic, Ruhan Dusunsel, Marc Fila, Anna Jander, Linda Koster-Kamphuis, Gregor Novljan, Pedro J. Ortega, Fabio Paglialonga, Maria T. Saravo, Constantinos J. Stefanidis, Christoph Aufricht, Kitty J. Jager, Franz Schaefer

**Affiliations:** 1 Division of Pediatric Nephrology and Gastroenterology, Medical University of Vienna, Vienna, Austria; 2 Department of Medical Informatics, Amsterdam Public Health research institute, ESPN/ERA-EDTA Registry and ERA-EDTA Registry, Amsterdam UMC, University of Amsterdam, Meibergdreef 9, Amsterdam, The Netherlands; 3 Pediatric Nephrology Unit, Bordeaux University Hospital, Bordeaux, France; 4 Department of Paediatric Nephrology, Emma Children’s Academic Medical Center, Amsterdam UMC, University of Amsterdam, Meibergdreef 9, Amsterdam, The Netherlands; 5 Pediatric Nephrology Unit, Hospital ‘La Paz’, Madrid, Spain; 6 Department of Nephrology, University Medical Center Ljubljana, Ljubljana, Slovenia; 7 Department of Pediatric Nephrology, Erciyes University Medical Faculty, Kayseri, Turkey; 8 Department of Pediatric Nephrology, Montpellier University Hospital, Montpellier, France; 9 Department of Pediatrics, Immunology and Nephrology, Polish Mothers Memorial Hospital Research Institute, Łódź, Poland; 10 Department of Pediatric Nephrology, Amalia Children’s Hospital Radboud University Medical Center, Nijmegen, The Netherlands; 11 Pediatric Nephrology Department, Children’s Hospital, University Medical Centre Ljubljana, Ljubljana, Slovenia; 12 Department of Pediatric Nephrology, Hospital Universitari La Fe, Valencia, Spain; 13 Pediatric Nephrology and Dialysis Unit, Fondazione IRCCS Ca’Granda Ospedal Maggiore Policlinico, Milan, Italy; 14 Nephrology and Dialysis Unit, Santobono Children’s Hospital, Naples, Italy; 15 Department of Nephrology, P&A Kyriakou Children’s Hospital, Athens, Greece; 16 Department of Pediatric Nephrology, University Children’s Hospital, Heidelberg, Germany

**Keywords:** Arteriovenous fistula, Central venous catheter, End-stage renal disease in children, Renal replacement therapy, Access to transplantation

## Abstract

**Background:**

Current guidelines advocate use of arteriovenous fistula (AVF) over central venous catheter (CVC) for children starting hemodialysis (HD). European data on current practice, determinants of access choice and switches, patient survival, and access to transplantation are limited.

**Methods:**

We included incident patients from 18 European countries who started HD from 2000 to 2013 for whom vascular access type was reported to the ESPN/ERA-EDTA Registry. Data were evaluated using descriptive statistics, logistic and Cox regression models, and cumulative incidence competing risk analysis.

**Results:**

Three hundred ninety-three (55.1%) of 713 children started HD with a CVC and were more often females, younger, had more often an unknown diagnosis, glomerulonephritis, or vasculitis, and lower hemoglobin and height-SDS at HD initiation. AVF patients were 91% less likely to switch to a second access, and two-year patient survival was 99.6% (CVC, 97.2%). Children who started with an AVF were less likely to receive a living donor transplant (adjusted HR, 0.30; 95% CI, 0.16–0.54) and more likely to receive a deceased donor transplant (adjusted HR, 1.50; 95% CI, 1.17–1.93), even after excluding patients who died or were transplanted in the first 6 months.

**Conclusions:**

CVC remains the most frequent type of vascular access in European children commencing HD. Our results suggest that the choice for CVC is influenced by the time of referral, rapid onset of end-stage renal disease, young age, and an expected short time to transplantation. The role of vascular access type on the pattern between living and deceased donation in subsequent transplantation requires further study.

**Electronic supplementary material:**

The online version of this article (10.1007/s00467-018-4129-6) contains supplementary material, which is available to authorized users.

## Introduction

European Best Practice Guidelines state minimizing dialysis time and performing kidney transplantation as early as possible as important treatment goals in the care of children [1–3]. However, pre-emptive transplantation is not always feasible, and dialysis is initiated in the majority of children starting renal replacement therapy (RRT) [4, 5].

Vascular access options for hemodialysis (HD) are arteriovenous fistulas (AVF), arteriovenous grafts (AVG), or central venous catheters (CVC). The Kidney Disease Outcome Quality Initiative Guidelines recommend using AVF as permanent access for most children on maintenance HD [6]. International initiatives, like the “International Pediatric Fistula First Initiative,” propagate an increased use of AVF instead of CVC [7–9].

Despite these recommendations, the percentage of CVC is still high and even seems to be increasing during recent years in the United States (USA) [10]. However, due to small patient numbers in pediatric dialysis units, there are limited data on current vascular access practices and the clinical course of pediatric patients commencing RRT on HD in Europe.

We therefore aimed to (i) analyze current practice in European countries with respect to vascular access in pediatric HD, (ii) evaluate differences in patient characteristics and clinical course, and (iii) investigate the association of first vascular access type for HD with access to renal transplantation and patient survival on RRT in children. To this end, we used data from the population-based European Society for Pediatric Nephrology (ESPN)/European Renal Association-European Dialysis and Transplant Association (ERA-EDTA) Registry.

## Methods

### Data collection

Thirty-eight European countries collect and provide individual patient data to the ESPN/ERA-EDTA Registry. A detailed description of the Registry can be found elsewhere [11]. For the current study, we included data of all patients starting RRT on HD from 1 January 2000 to 31 December 2013 for whom the vascular access type was reported (Online Resource 1). Data were obtained for the following variables: date of birth, sex, primary renal disease (PRD), start date of HD, date of follow-up measurements, types and dates of switches of treatment modality, date and cause of death, date of end of follow-up, vascular access type, hemoglobin, prescription of erythropoiesis-stimulating agent (yes/no), height and height standard deviation scores (SDS) at start of HD, and donor type for patients receiving a renal transplant.

### Definition of variables

Type of vascular access was coded as “AVF” (arteriovenous fistula), “AVG” (arteriovenous graft), or “CVC” (central venous catheter). Because of the low numbers of AVGs (*n* = 19), we excluded these patients from the analyses. Treatment modality changes and events were categorized as “switch of vascular access” (i.e., need for or transition to a second vascular access), “peritoneal dialysis” (PD), “recovery of renal function,” “renal transplantation” (TX) stratified by donor source, i.e., “living donor” (LD) and “deceased donor“(DD), or “death.” We defined age groups (< 6 years, 6 < 12 years, 12 < 16 years ≥ 16 years) and categorized the PRD according to the ERA-EDTA PRD codes for children [12]. Height SDS were calculated using recent national or European height-for-age charts [13].

### Statistical analysis

Data are shown as median and interquartile range (IQR) for continuous variables and as percentages for categorical variables. We used descriptive statistics to evaluate the differences in clinical characteristics between patients starting on AVF or CVC. To estimate differences in height SDS and hemoglobin levels adjusted for age, sex, PRD, and country, linear regression was used.

Odds ratios (ORs) for the likelihood of receiving an AVF were calculated for subgroups of patients, performing unadjusted and adjusted hierarchical logistic regression analysis (adjusted for sex, age group, and PRD) using a random intercept for country, thereby taking into account the variation in vascular access use across countries.

The unadjusted cumulative incidence competing risk (CICR) analysis was performed to estimate the two-year risk of vascular access switch [14], while Cox regression was used to estimate the likelihood of switching adjusted for potential confounders. Patients were followed from the start of HD until the end of the study period (31 December 2013), switch of RRT modality (LD or DD renal transplantation, or PD), switch to a second vascular access, and death or recovery of renal function, whichever occurred first.

Differences in overall access to transplantation and mortality within 2 years after HD initiation were analyzed using a Cox regression model with country as a random effect (correcting for clustered data within a country) and adjusted for potential confounders.

We performed several sensitivity analyses. As the use of AVF is less likely in young children, we repeated our analyses excluding patients under the age of 6 years at HD initiation. To test whether our results were representative for Europe and not merely reflect local practices, we repeated all analyses excluding patients from France (58% of patients).

Statistical analyses were performed in SAS 9.4 (SAS Institute, Inc. Cary, NC, USA). *p* values < 0.05 were considered statistically significant.

## Results

### Patient characteristics and determinants of first vascular access

A total number of 4619 patients commenced HD, and data on vascular access from 713 (15.4%) children was reported (Online Resource 1). Patients with available data on vascular access had a slightly higher median age at start of RRT than those without (14.2; IQR, 10.4–17.0 vs. 13.0; IQR, 8.6–16.0 years), while sex and PRD distribution was similar in both groups.

**Fig. 1:**
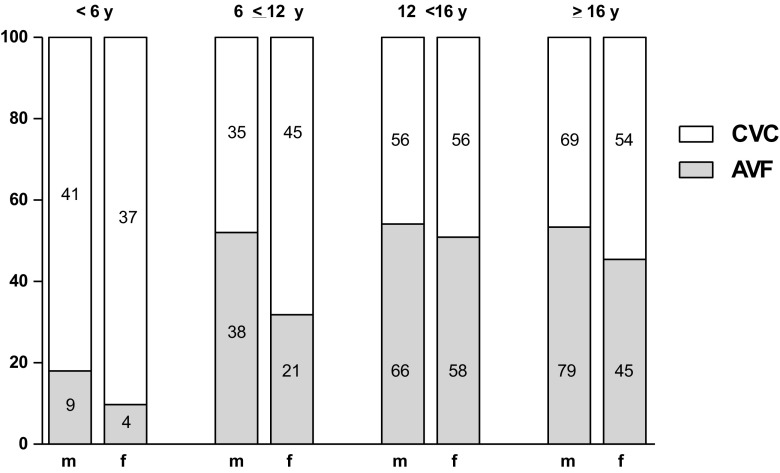
Vascular access at start of hemodialysis stratified by age group and sex. Patient numbers are presented in each bar. *CVC* central venous catheter, *AVF* arteriovenous fistula, *y* year, *m* male, *f* female

AVF was the first vascular access type in 320 patients (44.9%) and CVC in 393 patients (55.1%) (Table 1). Patients who received an AVF were significantly older when commencing HD, less often females, and had more often congenital anomalies of the kidney and urinary tract (CAKUT) as PRD than those with a CVC, while CVC patients more often presented with missing/unknown diagnoses (AVF, 15.3% vs. CVC, 23.9%). In Fig. 1, distributions of AVF and CVC stratified by age and sex are reported, and potential determinants of the first vascular access are depicted in Table 2.

**Table 1:** Demographic and clinical characteristics of patients at the start of hemodialysis

		Total	AVF	CVC	*p* value
Patients	*n* (%)	713	320 (44.9%)	393 (55.1%)	
Age (years)	Median (IQR)	14.2 (10.4–17.0)	14.7 (12.3–17.1)	13.6 (7.4–16.8)	< 0.001
Age group	*n* (%)				< 0.001
< 6 years		91 (12.8)	13 (4.1)	78 (19.9)	
6 < 12 years		139 (19.5)	59 (18.4)	80 (20.4)	
12–16 years		236 (33.1)	124 (38.8)	112 (28.5)	
> 16 years		247 (34.6)	124 (38.8)	123 (31.3)	
Sex (female)	*n* (%)	320 (44.9)	128 (40.0)	192 (48.9)	0.02
Primary renal disease	*n* (%)				< 0.001
Glomerulonephritis		178 (25.0)	74 (23.1)	104 (26.5)	
CAKUT		210 (29.5)	117 (36.6)	93 (23.7)	
Cystic kidney disease		52 (7.3)	28 (8.8)	24 (6.1)	
Hereditary nephropathy		64 (9.0)	29 (9.1)	35 (8.9)	
Ischemic renal failure		8 (1.1)	4 (1.3)	4 (1.0)	
Hemolytic uremic syndrome		27 (3.8)	13 (4.1)	14 (3.6)	
Metabolic disorders		13 (1.8)	4 (1.3)	9 (2.3)	
Vasculitis		18 (2.5)	2 (0.6)	16 (4.1)	
Missing/unknown diagnosis		143 (20.1)	49 (15.3)	94 (23.9)	
eGFR (ml/min/1.73 m^2^)	Median (IQR), *n*	8.0 (5.8–10.6), 499	8.2 (6.3–11.0), 226	7.8 (5.5–9.9), 273	0.03
Height-SDS	Median (IQR), *n*	− 1.39 (− 2.33;− 0.45), 522	− 1.35 (− 2.09;− 0.32), 230	− 1.49 (− 2.64; − 0.49), 292	0.03
Hemoglobin (g/dl)	Median (IQR), *n*	9.5 (8.2–11.0), 429	10.4 (9.0–11.8), 182	9.1 (7.7–10.4), 247	< 0.001
ESA (yes)	*n* (%), *n*	349 (89.0), 392	156 (88.1), 177	193 (89.8), 215	0.61

*AVF* arteriovenous fistula, *CVC* central venous catheter, *CAKUT* congenital anomalies of the kidney and urinary tract, *eGFR* estimated glomerular filtration rate, *SDS* standard deviation score, *ESA* erythropoiesis stimulating agent

**Table 2:** Likelihood of receiving an AVF at start of hemodialysis (unadjusted and adjusted odds ratios and confidence intervals)

Variables	OR unadjusted (95% CI)	*p* value	OR adjusted (95% CI)	*p* value
Sex^1^				
Female	0.69 (0.51–0.93)	0.02	0.67 (0.49–0.92)	0.01
Male (reference)	1.00		1.00	
Age groups^2^				
< 6 years	0.16 (0.08–0.23)	< 0.001	0.16 (0.08–0.30)	< 0.001
6 < 12 years	0.73 (0.48–1.13)	0.16	0.75 (0.49–1.16)	0.20
12–16 years	1.09 (0.75–1.57)	0.66	1.12 (0.77–1.63)	0.54
> 16 years (reference)	1.00		1.00	
Primary renal disease^3^				
Glomerulonephritis	0.53 (0.35–0.80)	0.003	0.51 (0.33–0.79)	0.002
CAKUT (reference)	1.00		1.00	
Cystic kidney disease	0.81 (0.44–1.51)	0.51	0.81 (0.43–1.55)	0.53
Hereditary nephropathy	0.63 (0.36–1.12)	0.12	0.75 (0.40–1.39)	0.36
Ischemic renal failure	0.76 (0.18–3.16)	0.71	0.81 (0.18–3.57)	0.78
Hemolytic uremic syndrome	0.68 (0.30–1.54)	0.36	0.82 (0.35–1.95)	0.66
Metabolic disorders	0.33 (0.10–1.12)	0.08	0.43 (0.12–1.55)	0.20
Vasculitis	0.09 (0.02–0.39)	0.002	0.08 (0.02–0.38)	0.001
Missing/unknown diagnosis	0.42 (0.27–0.67)	< 0.001	0.39 (0.24–0.64)	< 0.001

^1^Adjusted for age at start of HD and PRD; ^2^Adjusted for sex; ^3^Adjusted for sex and age at start of HD

*AVF* arteriovenous fistula, *CI* confidence interval, *OR* odds ratio, *CAKUT* congenital anomalies of the kidney and urinary tract, *PRD* primary renal disease

Hemoglobin levels at the start of HD were significantly higher in AVF patients. This difference remained statistically significant after adjustment for age, sex, PRD, and country (AVF, 10.2 g/dl and CVC, 9.1 g/dl (95% CI, 8.6–9.7); *p* < 0.001). When commencing HD, AVF patients were significantly taller than CVC patients (adjusted mean height SDS for AVF, − 1.29 and CVC, − 1.58 (95% C, − 1.95 to − 1.20); *p* = 0.049).

### Clinical course and switches of vascular access

**Fig. 2:**
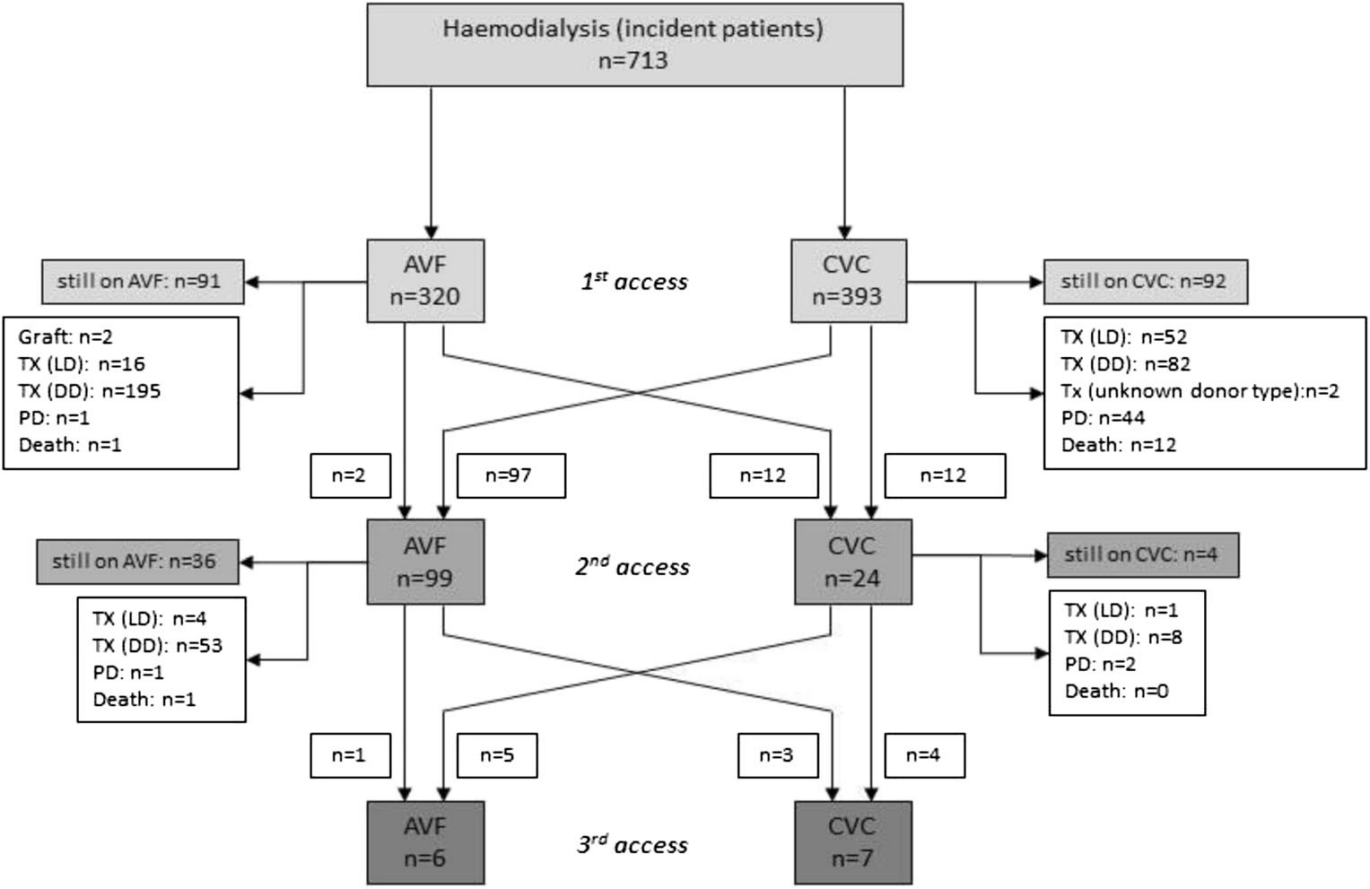
Flow diagram of 713 incident hemodialysis (HD) patients and their different treatment modalities and switch to another vascular access during their follow-up time. Total follow-up time was 1618 patient years (AVF, 769 patient years; CVC, 849 patient years). During follow-up, the overall crude rate of switching was 113 per 1000 patient years at risk (AVF, 31.2 per 1000 patient years; CVC, 187.4 per 1000 patient years). *AVF* arteriovenous fistula, *CVC* central venous catheter, *PD* peritoneal dialysis, *TX (LD)* transplantation (living donor), *TX (DD)* transplantation (deceased donor)

**Fig. 3:**
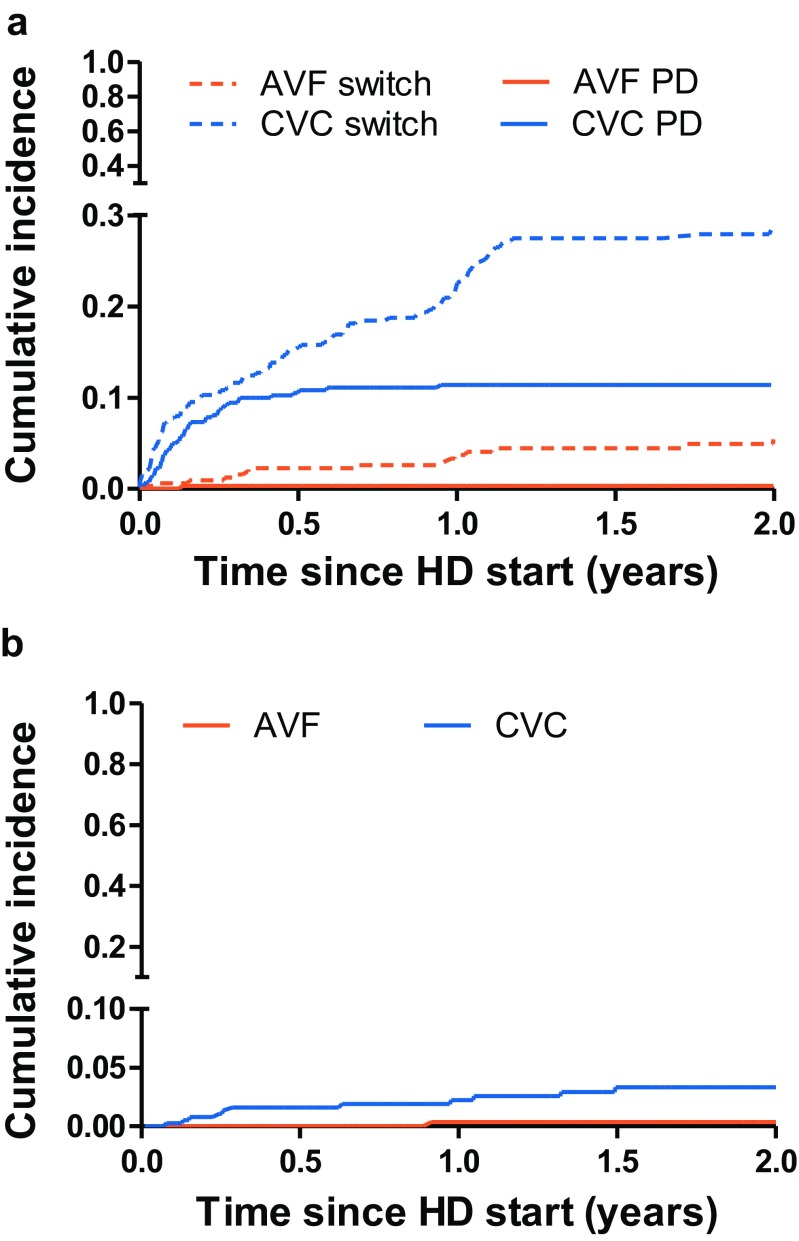
Cumulative incidence curves for **a** switch of first access or change to PD or **b** patient mortality stratified by first vascular access. *HD* hemodialysis, *AVF* arteriovenous fistula, *CVC* central venous catheter, *PD* peritoneal dialysis

The clinical course of patients starting HD with AVF and CVC is presented in a flow diagram showing absolute numbers (Fig. 2) and in a two-year cumulative incidence competing risk plot (Fig. 3a). Median time on first vascular access was 1.0 (IQR, 0.6–1.6) years for patients starting with an AVF and 0.5 (IQR, 0.2–1.0) years for those who started with a CVC (*p* < 0.001). Most CVC patients who received a second vascular access switched to an AVF (97/109).

After 2 years, 28.8% of AVF and 17.7% of CVC patients were still treated with their initial vascular access type, whereas 5.5% and 29.0% of AVF and CVC patients received a second vascular access, respectively (Fig. 3a). Similar results were found after excluding patients under the age of 6 years.

Patients who started with an AVF were 91% less likely to switch to a second vascular access as compared to those who started with a CVC (adjusted hazard ratio (aHR), 0.09; 95% CI, 0.05–0.16). Similar results were obtained when excluding children younger than 6 years of age (aHR, 0.09; 95% CI, 0.05–0.15) and after excluding patients from France (aHR, 0.13; 95% CI, 0.07–0.26).

### Patient survival

After a median follow-up of 0.78 (IQR, 0.25–1.33) years, 14 patients died (1 AVF; 13 CVC), resulting in a two-year patient survival of 99.6% for AVF and 96.7% for CVC patients (Fig. 3b). Cardiovascular disease was the cause of death in 4 patients (29%), infections in 2 (14%), the cause of death was unknown for 2 (14%) patients, and 6 patients (43%) died from other causes.

### Access to renal transplantation

Overall transplantation rates (combining LD and DD) after 2 years were 67.4% for patients who started with an AVF and 55.6% for those who started with a CVC. After adjustment for age at start of RRT, sex, PRD, and country, the likelihood of receiving a renal transplant within 2 years was not significantly different (Table 3). Similar results were obtained after excluding patients younger than 6 years (aHR, 1.03; 95% CI, 0.81–1.30), who received a renal transplant in the second year after commencing HD (aHR, 0.92; 95% CI, 0.69–1.21), and after excluding French patients (aHR, 1.06; 95% CI, 0.72–1.55).

**Table 3:** Two-year access to transplantation for patients with different first vascular access types (unadjusted and adjusted hazard ratios [HR] for AVF vs. CVC)

	Unadjusted HR (95% CI)	Adjusted HR (95% CI)
Overall	1.27 (1.03–1.57)	1.13 (0.90–1.41)
TX < 1 year since HD start	1.11 (0.85–1.44)	0.92 (0.69–1.21)
TX from living donor*	0.41 (0.23–0.74)	0.30 (0.16–0.54)
TX from deceased donor*	1.61 (1.27–2.04)	1.50 (1.17–1.93)

*AVF* arteriovenous fistula, *CVC* central venous catheter, *HR* hazard ratio, *CI* confidence interval, *TX* transplantation, *HD* hemodialysis

*Source of TX (deceased donor or living donor) not provided in 2 patients

**Fig. 4:**
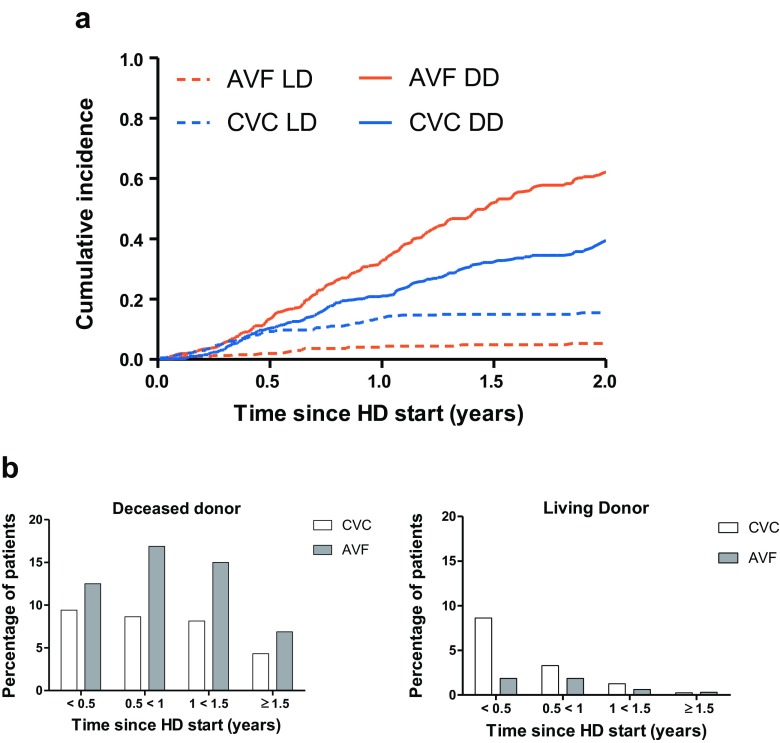
**a** Cumulative incidence for deceased donor (DD) or living donor (LD) transplantation stratified by first vascular access. **b** Percentage of patients receiving a kidney from a DD or LD stratified by vascular access type and time period since HD start. *HD* hemodialysis, *AVF* arteriovenous fistula, *CVC* central venous catheter, *LD* living donor, *DD* deceased donor

However, transplantation rates in patients started on AVF and CVC differed substantially with respect to donor type (Table 3). Patients who started with an AVF were far more likely to receive a kidney from a DD (62.1%), while CVC patients more often received a kidney from a LD (Fig. 4a). The timing of these transplantations was different. Most LD transplantations occurred in the first 6 months after initiating HD, while the proportion of DD transplantations was relatively stable and persistently lower in CVC patients (Fig. 4b). After excluding patients who were transplanted or died in the first 6 months, the likelihood of DD transplantation was still significantly higher in the AVF group compared with the CVC group irrespective of country, age, sex, and PRD (aHR, 1.71; 95% CI, 1.26–2.31).

## Discussion

This is the first study providing data on vascular access of incident pediatric HD patients and their clinical course including subsequent transplantation from several European countries. In European practice, more children started HD on a CVC instead of an AVF. Current knowledge, such as barriers for successful placement of an AVF in a child (patient being too small, AVF not created in time, scheduled transplantation), as well as a more stable clinical course with less switches of vascular access with AFV were confirmed. For the first time, data on HD vascular access were related to data on subsequent transplantation: While overall transplantation rates were equal, there were major differences in donor type and timing of transplantation between children initiating HD with AVF or CVC. Central venous catheter patients more often received LD transplants than AVF patients, mostly during the first 6 months after commencing HD. By contrast, CVC patients were less likely than AVF patients to receive a transplant from a DD within the first 2 years of dialysis, even when accounting for differences in age, sex, PRD, and country of residence.

The observed high proportion of CVC as first vascular access (56%) in European practice is in contrast to recommendations in recent initiatives and guidelines, but in keeping with previous studies [10, 15, 16]. Some large US studies even reported a CVC usage of 80–90% in incident HD children [10, 17, 18]. In concordance with previous studies, we found that patients who started HD on a CVC were significantly younger than patients with an AVF, mainly because most pre-school and pre-pubertal children started HD with a CVC [15, 17, 19]. Interestingly, more than 10% of those who started HD at an age under 6 years started with an AVF, demonstrating the option of creating an AVF in these young children [20]. As previously reported, also in our study, female patients were at higher risk for receiving a CVC which underlines the need for future studies of sex-specific factors in the decision-making process in creation of vascular accesses [21–23]. Moreover, in keeping with data reported for the USA, patients who started with a CVC were more often diagnosed with a PRD associated with rapid renal function decline or with delayed diagnosis, factors that might challenge pre-dialysis care [24]. Pre-dialysis care represents another well-known determinant for the choice of vascular access in HD patients, and late referral has been associated with a lower use of AVF [25, 26]. Indeed, parameters for outcome of pre-dialysis care, such as severity of anemia and stunted growth at onset of RRT, were both associated with a reduced likelihood of receiving an AVF [27–29]. Taken together, these data from the ESPN/ERA-EDTA Registry successfully confirm previous reports on barriers for AVF in the largest European population.

With regard to the clinical course on HD, CVC patients were far more likely to switch to an AVF or to PD as compared with AVF patients switching to CVC or PD. Data on infections or thromboembolic complications as potential cause for failing of access are not provided to the Registry. As most of these switches occurred within 3 months, however, we speculate that the majority switches from CVC to AVF constituted a delayed decision to create an AVF [26, 30, 31]. Thus, CVC was likely provided in a relevant proportion of incident HD patients as a “bridging therapy” until a permanent dialysis access was created.

Use of the ESPN/ERA-EDTA Registry allows for the first time to relate the choice of vascular HD access type to subsequent renal transplantation in a large European pediatric population. AVF patients and their parents typically have sufficient time and information to consider all RRT options before start of HD, including screening for potential LD for pre-emptive transplantation [25, 26, 28, 32]. Thus, non-pre-emptive LD was rare in the cohort of AVF patients. By contrast, CVC may be chosen as first treatment modality for elective as well as non-elective reasons [10, 15, 17–19, 33]. These patients include the ones in whom LD was planned, but who needed RRT before LD TX was completed, suggesting again that CVC is offered as a “bridging therapy.” Indeed, in a US study, CVC patients in whom transplantation was scheduled had a shorter time to transplantation than AVF patients [17]. In the Registry, the likelihood of receiving a LD transplant was significantly higher for the CVC group, especially in the first 6 months after HD initiation.

Interestingly, CVC patients were less likely than AVF patients to receive a transplant from a DD within the first 2 years of dialysis, even when accounting for differences in age, sex, PRD, and country of residence. As the absolute proportion of CVC patients receiving a LD is only 15.5%, anticipated LD cannot be the only reason why the access to DD TX was reduced in the CVC compared to the AVF group. Moreover, after excluding patients who were transplanted or died within the first 6 months, AVF patients still had a consistently higher likelihood of DD transplantation than CVC patients. By this time, expected LD transplants have already been performed, and patients with poor initial clinical status should have stabilized. We may speculate that other factors might contribute to the lower likelihood of receiving DD transplantation in CVC patients, such as severity and complexity of patients’ kidney disease, extra-renal comorbidities, ethnic disparities, and local center policies, but this requires further study.

The most important strength of our study is that we used data from the ESPN/ERA-EDTA Registry, a meta-registry that prospectively collects population-based data from European national registries. While sensitivity analysis suggests that our cohort is representative for the European pediatric HD population, it should be noted that vascular access type was only reported for 15% of all incident HD patients included in the Registry. As a strength, longitudinal data regarding changes in treatment, subsequent transplantation, and patient survival were available, which enabled us to follow the course of HD treatment. Further limitations of our work include the lack of data on reasons to choose CVC or AVF, referral date, catheter type, fistula creation date, infections and comorbidities, and on local policies and center characteristics, which will require further research in independent studies.

In conclusion, our study demonstrates that notwithstanding the fistula-first initiative CVC still remains the major mode of vascular access in European children despite a higher need for switch to alternate access [9]. This practice is explained in part by CVC being the only option in infants and its use as a bridging therapy to living-related transplantation. Referral timing, CKD progression rate, and the clinical condition at start of dialysis might be additional determinants affecting the choice of vascular access in children. While overall transplantation rates were equal for both vascular access types within the 2-years observation period, CVC patients and AVF patients markedly differed in the likelihood to receive their transplants from LD versus DD. This relationship between choice of hemodialysis vascular access type and donor pattern of subsequent transplantation requires further study.

## Electronic supplementary material

ESM 1 (DOCX 13.3 kb)

## References

[1] Friedewald JJ, Reese PP (2012). The kidney-first initiative: what is the current status of preemptive transplantation?. Adv Chronic Kidney Dis.

[2] Kasiske BL, Snyder JJ, Matas AJ, Ellison MD, Gill JS, Kausz AT (2002). Preemptive kidney transplantation: the advantage and the advantaged. J Am Soc Nephrol.

[3] European best practice guidelines for renal transplantation. Section IV (2002). Long-term management of the transplant recipient. IV.11 Paediatrics (specific problems). Nephrol Dial Transplant.

[4] Harambat J, van Stralen KJ, Kim JJ, Tizard EJ (2012). Epidemiology of chronic kidney disease in children. Pediatr Nephrol.

[5] Hogan J, Audry B, Harambat J, Dunand O, Garnier A, Salomon R, Ulinski T, Macher MA, Couchoud C (2015). Are there good reasons for inequalities in access to renal transplantation in children?. Nephrol Dial Transplant.

[6] Clinical practice recommendation 8 (2006). Vascular access in pediatric patients. Am J Kidney Dis 48 Suppl.

[7] Chand DH, Swartz S, Tuchman S, Valentini RP, Somers MJ (2017). Dialysis in children and adolescents: the pediatric nephrology perspective. Am J Kidney Dis.

[8] Chand DH, Geary D, Patel H, Greenbaum LA, Nailescu C, Brier ME, Valentini RP (2015). Barriers, biases, and beliefs about arteriovenous fistula placement in children: a survey of the International Pediatric Fistula First Initiative (IPFFI) within the Midwest Pediatric Nephrology Consortium (MWPNC). Hemodial Int.

[9] Chand DH, Valentini RP (2008). International pediatric fistula first initiative: a call to action. Am J Kidney Dis.

[10] Mak RH, Warady BA (2013). Dialysis: vascular access in children--arteriovenous fistula or CVC?. Nat Rev Nephrol.

[11] ESPN/ERA-EDTA Registry website. http://www.espn-reg.org/

[12] ERA-EDTA Registry (2017) ERA-EDTA Registry Annual Report 2015

[13] Bonthuis M, van Stralen KJ, Verrina E, Edefonti A, Molchanova EA, Hokken-Koelega AC, Schaefer F, Jager KJ (2012). Use of national and international growth charts for studying height in European children: development of up-to-date European height-for-age charts. PLoS One.

[14] Noordzij M, Leffondre K, van Stralen KJ, Zoccali C, Dekker FW, Jager KJ (2013). When do we need competing risks methods for survival analysis in nephrology?. Nephrol Dial Transplant.

[15] Hayes WN, Watson AR, Callaghan N, Wright E, Stefanidis CJ, European Pediatric Dialysis Working G (2012). Vascular access: choice and complications in European paediatric haemodialysis units. Pediatr Nephrol.

[16] Shroff R, Sterenborg RB, Kuchta A, Arnold A, Thomas N, Stronach L, Padayachee S, Calder F (2016). A dedicated vascular access clinic for children on haemodialysis: two years’ experience. Pediatr Nephrol.

[17] Fadrowski JJ, Hwang W, Neu AM, Fivush BA, Furth SL (2009). Patterns of use of vascular catheters for hemodialysis in children in the United States. Am J Kidney Dis.

[18] North American Pediatric Renal Trialys and Collaborative Studies (2011) NAPRTCS Annual Report. http://emmes.com/study/ped/annlrept/annualrept2011.pdf

[19] Ma A, Shroff R, Hothi D, Lopez MM, Veligratli F, Calder F, Rees L (2013). A comparison of arteriovenous fistulas and central venous lines for long-term chronic haemodialysis. Pediatr Nephrol.

[20] Karava V, Jehanno P, Kwon T, Deschenes G, Macher MA, Bourquelot P (2017). Autologous arteriovenous fistulas for hemodialysis using microsurgery techniques in children weighing less than 20 kg. Pediatr Nephrol.

[21] Hogan J, Couchoud C, Bonthuis M, Groothoff JW, Jager KJ, Schaefer F, Van Stralen KJ, Registry EE-E (2016). Gender disparities in access to pediatric renal transplantation in Europe: data from the ESPN/ERA-EDTA Registry. Am J Transplant.

[22] Casey JR, Hanson CS, Winkelmayer WC, Craig JC, Palmer S, Strippoli GF, Tong A (2014). Patients’ perspectives on hemodialysis vascular access: a systematic review of qualitative studies. Am J Kidney Dis.

[23] Noordzij M, Jager KJ, van der Veer SN, Kramar R, Collart F, Heaf JG, Stojceva-Taneva O, Leivestad T, Buturovic-Ponikvar J, Benitez Sanchez M, Moreso F, Prutz KG, Severn A, Wanner C, Vanholder R, Ravani P (2014). Use of vascular access for haemodialysis in Europe: a report from the ERA-EDTA Registry. Nephrol Dial Transplant.

[24] O’Shaughnessy MM, Montez-Rath ME, Zheng Y, Lafayette RA, Winkelmayer WC (2016). Differences in initial hemodialysis vascular access use among glomerulonephritis subtypes in the United States. Am J Kidney Dis.

[25] Erickson KF, Mell M, Winkelmayer WC, Chertow GM, Bhattacharya J (2015). Provider visits and early vascular access placement in maintenance hemodialysis. J Am Soc Nephrol.

[26] Smart NA, Dieberg G, Ladhani M, Titus T (2014) Early referral to specialist nephrology services for preventing the progression to end-stage kidney disease. Cochrane Database Syst Rev (6):CD007333 10.1002/14651858.CD007333.pub2 24938824

[27] Boehm M, Riesenhuber A, Winkelmayer WC, Arbeiter K, Mueller T, Aufricht C (2007). Early erythropoietin therapy is associated with improved growth in children with chronic kidney disease. Pediatr Nephrol.

[28] Boehm M, Winkelmayer WC, Arbeiter K, Mueller T, Aufricht C (2010). Late referral to paediatric renal failure service impairs access to pre-emptive kidney transplantation in children. Arch Dis Child.

[29] Kennedy SE, Bailey R, Kainer G (2012). Causes and outcome of late referral of children who develop end-stage kidney disease. J Paediatr Child Health.

[30] Lorenzo V, Martn M, Rufino M, Hernandez D, Torres A, Ayus JC (2004). Predialysis nephrologic care and a functioning arteriovenous fistula at entry are associated with better survival in incident hemodialysis patients: an observational cohort study. Am J Kidney Dis.

[31] Raithatha A, McKane W, Kendray D, Evans C (2010). Catheter access for hemodialysis defines higher mortality in late-presenting dialysis patients. Ren Fail.

[32] Cavanaugh KL, Wingard RL, Hakim RM, Elasy TA, Ikizler TA (2009). Patient dialysis knowledge is associated with permanent arteriovenous access use in chronic hemodialysis. Clin J Am Soc Nephrol.

[33] Watson AR, Hayes WN, Vondrak K, Ariceta G, Schmitt CP, Ekim M, Fischbach M, Edefonti A, Shroff R, Holta T, Zurowska A, Klaus G, Bakkaloglu S, Stefanidis CJ, Van de Walle J, European Paediatric Dialysis Working G (2013). Factors influencing choice of renal replacement therapy in European paediatric nephrology units. Pediatr Nephrol.

