# Supplementary material for: Hemodialysis vascular access and subsequent transplantation: a report from the ESPN/ERA-EDTA Registry
Source: Pediatr Nephrol. 2018 Dec 26;34(4):713–21. doi: 10.1007/s00467-018-4129-6 (PMC6394682; doi:10.1007/s00467-018-4129-6)
Supplement: Supplementary file 1 — (DOCX 13.3 kb) [file 467_2018_4129_MOESM1_ESM.docx]

**Online Resource 1 Incident HD patients starting RRT from 01-01-2000 to 31-12-2013**

| **Country** | **Total patients starting RRT on HD**  n | **Patients with reported access type**  n (%) |
| --- | --- | --- |
| Total | 4619 | 713 (15.4%) |
| Albania | 10 | 1 (10.0) |
| Austria | 135 | 22 (16.3) |
| Belarus | 37 | 3 (8.1) |
| Belgium | 49 | 2 (4.1) |
| Finland | 66 | 4 (6.1) |
| France | 894 | 412 (46.1) |
| Hungary | 38 | 1 (2.6) |
| Italy | 275 | 180 (65.5) |
| Lithuania | 25 | 1 (4.0) |
| The Netherlands | 76 | 34 (44.7) |
| Norway | 62 | 2 (3.2) |
| Poland | 326 | 26 (8.0) |
| Portugal | 36 | 3 (8.3) |
| Russia | 373 | 1 (0.3) |
| Serbia | 74 | 9 (12.2) |
| Slovenia | 21 | 5 (23.8) |
| Slovakia | 41 | 4 (9.8) |
| Turkey | 192 | 3 (1.7) |
| Other countries* | 1889 | - |

* Including: Bosnia and Herzegovina, Bulgaria, Croatia, Czech Republic, Denmark, Estonia, Georgia, Greece, Iceland, Moldova, Montenegro, Romania, Spain, Sweden, Switzerland, Ukraine, and United Kingdom.

*HD* Haemodialysis, *RRT* Renal Replacement Therapy, *AVF* Arteriovenous Fistula, *CVC* Central Venous Catheter
